# Radiofrequency Catheter Ablation of Idiopathic Right Ventricular Outflow Tract Arrhythmias

**DOI:** 10.1016/s0972-6292(16)30585-x

**Published:** 2013-01-01

**Authors:** Naiara Calvo, Monique Jongbloed, Katja Zeppenfeld

**Affiliations:** 1 Department of Cardiology, Leiden University Medical Center, Leiden, The Netherlands; 2 Department of Anatomy and Embryology, Leiden University Medical Center, Leiden, The Netherlands

**Keywords:** ventricular arrhythmias, outflow tract, ICDs, premature ventricular contractions, ablation

## Abstract

Idiopathic ventricular arrhythmias (VA) consist of various subtypes of VA that occur in the absence of clinically apparent structural heart disease. Affected patients account for approximately 10% of all patients referred for evaluation of ventricular tachycardia (VT). Arrhythmias arising from the outflow tract (OT) are the most common subtype of idiopathic VA and more than 70-80% of idiopathic VTs or premature ventricular contractions (PVCs) originate from the right ventricular (RV) OT. Idiopathic OT arrhythmias are thought to be caused by adenosine-sensitive, cyclic adenosine monophosphate (cAMP) mediated triggered activity and, in general, manifest at a relatively early age. Usually they present as salvos of paroxysmal ventricular ectopic beats and are rarely life-threatening. When highly symptomatic and refractory to antiarrhythmic therapy or causative for ventricular dysfunction, ablation is a recommended treatment with a high success rate and a low risk of complications.

## Introduction

Idiopathic ventricular arrhythmias (VA) consist of various subtypes of VA that occur in the absence of clinically apparent structural heart disease. Affected patients account for approximately 10% of all patients referred for evaluation of ventricular tachycardia (VT) [1]. Arrhythmias arising from the outflow tract (OT) are the most common subtype of idiopathic VA and more than 70-80% of idiopathic VTs or premature ventricular contractions (PVCs) originate from the right ventricular (RV) OT [2]. Idiopathic OT arrhythmias are thought to be caused by adenosine-sensitive, cyclic adenosine monophosphate (cAMP) mediated triggered activity and, in general, manifest at a relatively early age. Usually they present as salvos of paroxysmal ventricular ectopic beats and are rarely life-threatening. When highly symptomatic and refractory to antiarrhythmic therapy or causative for ventricular dysfunction, ablation is a recommended treatment with a high success rate and a low risk of complications.

The aim of this review is to provide insights into the development and anatomy of the RVOT as related to arrhythmias and to describe the clinical characteristics and the current methods to localize and ablate these arrhythmias.

## Embryological development of the outflow tracts

Initially, the outlet portion of the heart is a single myocardial tube that connects to the aortic sac and the connecting pharyngeal arch arteries. Septation of the outlet portion structures takes place at three levels: 1. the level of the myocardial OT, 2. the level of the semilunar valves and 3. the level of the aortic sac that will divided into an aorta and a pulmonary trunk [3,4]. During development a significant lengthening of the right ventricular outflow tract occurs, whereas the left ventricular outflow tract (LVOT) remains relatively short, reflecting the right/left asymmetry. This is further substantiated by the fact that the putative left ventricle is one of the first compartments to be recognized in the embryonic heart, whereas the right ventricle is added to the heart during later developmental stages [5], including a marked lengthening of the sub-pulmonary myocardium characterized by a specific gene expression pattern [6].

**Figure 1:**
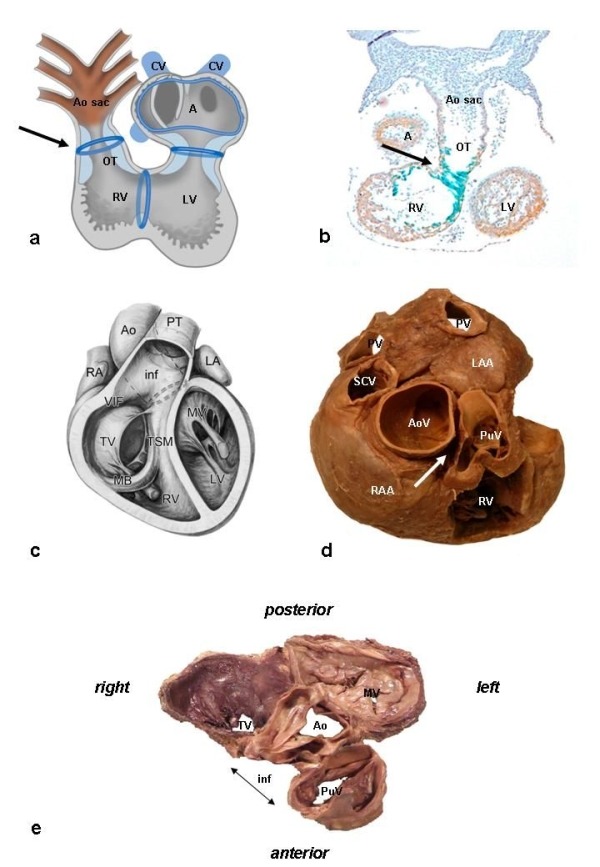
Embryology and Anatomy. Panel A: Schematic depiction of the heart tube during an early developing stage, after looping of the heart has been initiated. Several transitional zones, located in between the different cardiac segments, can be recognized in the heart based on histological characteristics as well as expression of molecular markers. These zones, indicated schematically in blue, will contribute to elements of the cardiac conduction system. The ventriculo-arterial transition, indicated by the arrow, is situated at the level of the outflow tract (OT) of the heart. In the adult heart, only part of these transitional zones can still be recognized, in elements of the definitive cardiac conduction system (not shown) ( reproduced with permission from Gittenberger-de Groot AC et al. James H. Moller , Julien I. E. Hoffman, eds. Pediatric Cardiovascular Medicine. Second ed.December 2011, Wiley-Blackwell). Panel B: Expression of the marker CCS-lacZ that can be found during embryonic heart development in elements of the cardiac conduction system and the transitional zones. Shown here is CCS-lacZ expression at the level of the OT (arrow) (reproduced with permission from Jongbloed et al. J Cardiovasc Electrophysiol 2004;15(3):349-355). Panel C: Anatomy of the outflow tract, anterior view, demonstrating the position of the great arteries in the normal heart. The aorta (Ao) is positioned right posteriorly in relation to the pulmonary trunk (PT). On the right side, the fibrous tissue of the tricuspid valve (TV) is separated from the fibrous tissue of the pulmonary valve by the posterior wall of the muscular infundibulum (inf) (reproduced with permission from Bartelings MM and Gittenberger-de Groot AC (1989) The outflow tract of the heart - embryologic and morphologic correlations. Int.J.Cardiol. 22, 289-300). Panel D: Superior view. The aorta has a central position and is wedged in between the atrioventricular orifices and appendages of the right atrium (RAA) and left atrium (LAA). The aortic valve (AoV) has an inferior position as compared to the pulmonary valve (PuV). Note that there is no continuity between the distal medial and posterior wall of the RVOT and the aorta (arrow). Panel E: The fibrous heart skeleton. Note the fibrous continuity between the aortic valve, mitral valve (MV) and tricuspid valve, whereas there is no continuity with the fibrous tissue of the pulmonary valve due the presence of the subpulmonary infundibulum. Other abbreviations: A: common atrium, AoS: aortic sac, CV: cardinal veins, LA: left atrium, LV: left ventricle, MB: moderator band, PV: pulmonary vein, RA: right atrium, RV right ventricle, SCV: superior caval vein, TSM: trabecula-septomarginalis.

During development and after looping of the primitive heart tube, several so-called "transitional zones" can be recognized, that are related to elements of the putative cardiac conduction system (Figure 1A,B), which show slow conducting properties as opposed to the remainder of the embryonic heart [7]. One of these transitional zones is found at the level of the myocardial OT. Several markers related to the developing cardiac conduction system have been described in these zones [8], including the Hyperpolarization-activated cyclic nucleotide-gated channel 4 (HCN4), that is responsible for the (I_f_) or funny current in the sino-atrial node [9]. The expression of HCN4 among other markers, including CCS-lacZ and MinK-lacZ [10,11], in particular areas of the developing heart may explain the occurrence of arrhythmias at specific predilection sites in the adult heart, including the RVOT. The developing cardiac conduction system thus covers a much broader area than the definitive adult cardiac conduction system, and although the definitive cause of idiopathic RVOT VA or ectopy has not been resolved to date, either a re-expression of an embryonic phenotype, or embryonic remnants of tissue may provide an explanation the arrhythmogenic potential of this area.

## Anatomy of the outflow tracts

During OT development the great arteries achieve their definitive relationship, with the aorta situated in a central position, right posterior of the pulmonary trunk. The RVOT thus courses anterior to the LVOT, from a rightward inferior to a leftward superior direction (Figure 1C). Both the LVOT and RVOT have their own morphological characteristics. The RVOT is characterized by the presence of a muscular sub-pulmonary infundibulum that forms a circular muscular tube below the pulmonary valve. Due to the length of this sub-pulmonary myocardium, the pulmonary valve has a more superior position as compared to the aortic valve. The pulmonary annulus is thus positioned superior and to the left of the aortic annulus and the pulmonary trunk continues leftward and divides in a right and left pulmonary artery, the right of which will course below the aortic arch [12].

The posterior wall of the sub-pulmonary infundibulum is situated between the tricuspid valve and the pulmonary valve. The proximal medial wall of the RVOT is formed by the anterior part of the interventricular septum and is separated from the inflow part by the trabecula septomarginalis that contains the right bundle branch (Figure 1C) [13]. Of importance, there is no continuity between the distal medial and posterior wall of the RVOT and the aorta. The term "septal" RVOT is therefore misleading (Figure 1D, arrow)

On the left side there is fibrous continuity between the aortic valve and the mitral valve (Figure 1E). The sub-aortic region is formed by the fibrous tissue of the aortic leaflet of the mitral valve, the membranous part of the ventricular septum, as well as by the muscular ventricular septum, and the anterior left ventricular wall. The fibrous tissues of the tricuspid valve, mitral valve and aortic valve are all part of the fibrous heart skeleton, whereas the pulmonary valve is positioned more anterosuperiorly and is usually not in fibrous continuity with the other valves. The bundle of His, in the normal heart the only myocardial bundle passing the annulus fibrosus, and thereafter runs in the lower edge of the membranous part of the ventricular septum, is situated on top of the muscular part of the ventricular septum, where is branches into a left and right bundle branch.

## Clinical characteristics

**Figure 2:**
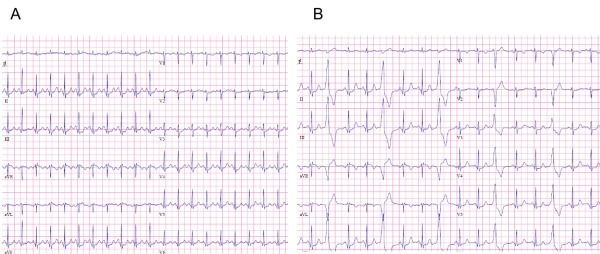
Suppression with exercise: 12 lead ECG during treadmill exercise testing is shown. Panel A: 12 lead ECG during exercise (at peak exercise after 8 minutes). Panel B: 12 lead ECG during the recovery phase (2 minutes) of exercise. Note the suppression of PVCs during exercise.

Patients usually present in their second to fifth decade [14] with symptomatic palpitations. Presyncope and light-headedness may be observed, but true syncope is infrequent (<10%). Although the arrhythmia may be sustained, it is usually characterized by repetitive bursts of nonsustained VT or frequent PVCs. Arrhythmias can often be provoked by exercise, caffeine and emotional stress. In women, who are more frequently affected, RVOT VA are commonly reported during the premenstrual and perimenopausal period and during pregnancy, suggesting hormonal flux as a trigger for RVOT arrhythmias [14,15]. However, in a significant number of patients, exercise suppresses the arrhythmia and VA arises during the recovery phase or during rest [16] (Figure 2).

In general, tachycardia from the OT shows a benign course. However, it has been reported that frequent PVCs and VTs can cause left ventricular (LV) dysfunction that can be reversed by suppression of VA with antiarrhythmic agents or radiofrequency catheter ablation [17,18].

It is important to distinguish idiopathic RVOT VA from VA caused by structural heart diseases, such as arrhythmogenic right ventricular dysplasia (ARVD), in which the RVOT may be one of the origins of potential life threatening VA [19]. A careful analysis of the 12-lead surface ECG, a detailed medical and family history,exercise testing and cardiac imaging may be warranted to establish the diagnosis.

Idiopathic RVOT VA typically occurs in patients with no structural heart disease. However, several MRI studies have reported subtle areas of focal thinning and segmental contraction abnormalities, as well as focal fatty infiltration [20-23]. In contrast, other studies showed no evidence of structural heart disease on MRI [24,25]. Therefore, to date, there is not conclusive evidence for structural abnormalities associated with truly "idiopathic" RVOT VA.

**Figure 3:**
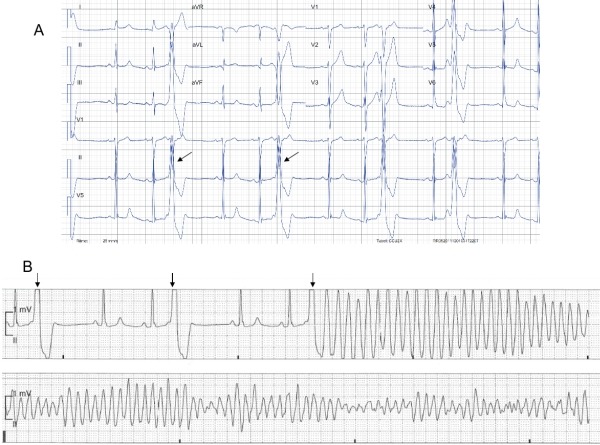
Panel A: Twelve-lead ECG of a patient with frequent, monomorphic PVCs with left bundle branch morphology and inferior axis, originating from RVOT. Panel B: Initiation of VF by the same PVC as recorded by a monitoring ECG of the patient. Note that the QRS morphology of the initiating PVCs was identical to that of the preceding isolated PVC (arrows).

Rarely, ectopy from the RVOT can trigger ventricular fibrillation and/or polymorphic VT (Figure 3) [26-28]. A shorter coupling interval (CI) to the preceding QRS complex, and a shorter cycle length (CL) during monomorphic VT, if present, have been found to predict the occurrence of VF or polymorphic VT [29]. In contrast, a more recent study [30] demonstrated that the prematurity index (PI) (defined as the ratio of the CI of the first VT beat or isolated PVC to the preceding R-R interval of the sinus cycle just before the VT or isolated PVC), but no the CI, was the only independent predictor for polymorphic VT; a PI value of <0.73 predicted occurrence of polymorphic VT with a sensitivity of 91% and a specificity of 44%.

## Arrhythmia mechanism

The majority of idiopathic OT arrhythmias are thought to be due to calcium-dependent triggered activity mediated through cAMP resulting in delayed afterdepolarizations [31,32]. Rapid burst pacing, isoproterenol infusion, and rarely aminophylline, calcium infusion, or atropine may facilitate arrhythmia induction [33]. These interventions lead to increased intracellular cAMP, which, via activation of the protein kinase A, increases the slow inward Ca2+ current and the calcium release from the sarcoplasmic reticulum through phosphorylation of the ryanodine receptor. Calcium released from the SR can activate the electrogenic Na+/Ca2+ exchanger, resulting in a transient inward current and delayed afterdepolarization [34,35].

## Localization of the site of origin

RVOT VAs often arise from the distal medial ("septal") and anterior surface [2,16,36-40]. However, a free wall focus has also been reported in 20-30% of patients undergoing radiofrequency (RF) ablation of RVOT VA [41].

The majority of "septal" and free wall VA originates from the distal RVOT. Yamashina et al. [42] have evaluated the distribution of successful ablation sites within the RVOT using a three-dimensional electroanatomical mapping system and demonstrated that 88.7% of successful ablation sites were located in the transitional-voltage zone beneath the pulmonary valve, with a mean vertical length of 8.1 mm. However, RVOT VAs arising from the pulmonary artery or near the bundle of His have also been described [43-46].

## The role of the 12-lead electrocardiogram

**Figure 4:**
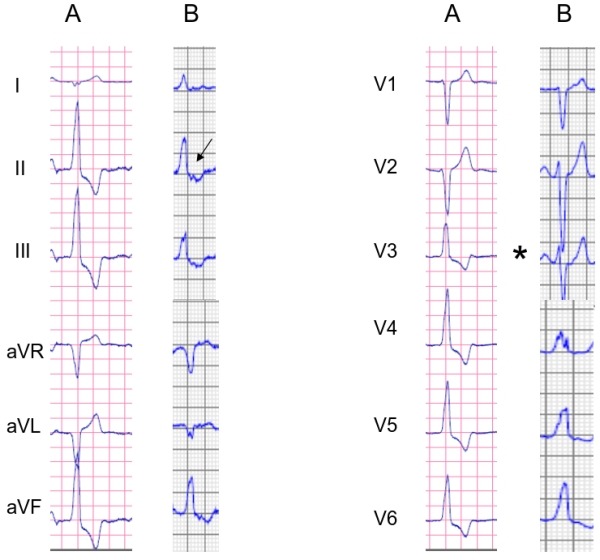
Typical ECG morphologies of 2 clinical arrhythmias originating from the RVOT. Panel A: Electrocardiogram of a tachycardia arising from the anterior "septal" RVOT. Note the QS complex in lead I, narrow QRS, absence of R-wave notching in the inferior leads, and R > S in lead V3. B) Electrocardiogram of a tachycardia arising from the free wall. Note the positive QRS in lead I, R-wave notching in the inferior leads (arrow), and R < S in lead V3 (*).

Ventricular arrhythmias originating from the RVOT typically demonstrate left bundle branch block morphology with an inferiorly directed frontal QRS axis. On the basis of pace-mapping, several electrocardiographic algorithms have been proposed to guide catheter ablation of ventricular arrhythmias originating from the RVOT [2,47-49]. A QS complex in frontal lead I suggests an anterior site, while an R or qR complex indicates a more posterior site of origin (Figure 4) [50-52].

**Figure 5:**
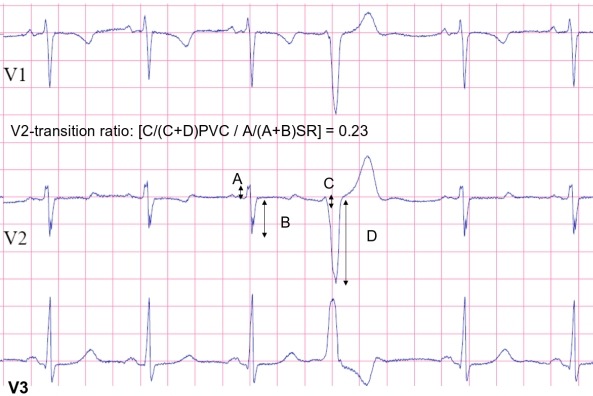
Precordial leads V1,V2 and V3 of sinus rhythm and a premature ventricular contraction (PVC) from the antero-"septal" region of right outflow tract, with R/S transition at lead V3. A: sinus rhythm R-wave amplitude (mV); B: A sinus rhythm S-wave amplitude (mV); C: PVC R-wave amplitude (mV); D: PVC S-wave amplitude (mV); The transition ratio was calculated with the following formula: [C/(C+D)PVC / A/(A+B)SR]. The V2 transition ratio was 0.23.

The precordial lead transition (defined as the first precordial lead with R≥S) moves leftward as the origin moves diagonally from the distal-posterior "septal" aspect of the RVOT to the proximal-anterior region. In OT VA with a precordial R/S transition in lead V3, a V2 transition ratio <0.6 and a precordial transition of the VA QRS later than the transition of the sinus rhythm (SR) QRS reliably predicted an RVOT origin (53) (Table 1) (Figure 5).

A wider QRS complex and notching of the R-wave in the inferior leads have been associated with free wall RVOT VA. In contrast, a "septal" origin leads to a simultaneous right- and left sided activation causing a relatively narrow QRS complex. An isoelectric or positive QRS complex in lead aVL is typically recorded if the site of origin is located in the proximal RVOT while a distal site of origin produces a negative QRS complex [50].

More recently, Zhang et al. [54] have developed a new ECG algorithm based on the location of successful ablation sites as identified by detailed 3D mapping using a non-contact-electroanatomical mapping system (Ensite system, Ensite array, St Jude Medical, St. Paul, MN). According to their observations, a transitional zone of ≥V4, a R wave duration index of less than 0.5, or a R/S amplitude index of less than 0.3 in the precordial leads V1 and V2 was highly predictive for an RVOT origin (Table 1). Although the precise localization of the site of origin based on ECG criteria is limited by the close anatomic relation of the LVOT, an inferior QRS axis and a very early precordial transition zone (RS ratio ≥1 in leads V1 or V2) is usually consistent with a LVOT origin (basal septum or aortic commissures) or with an origin from the LV epicardium [37,48].

## Mapping strategy

**Figure 6:**
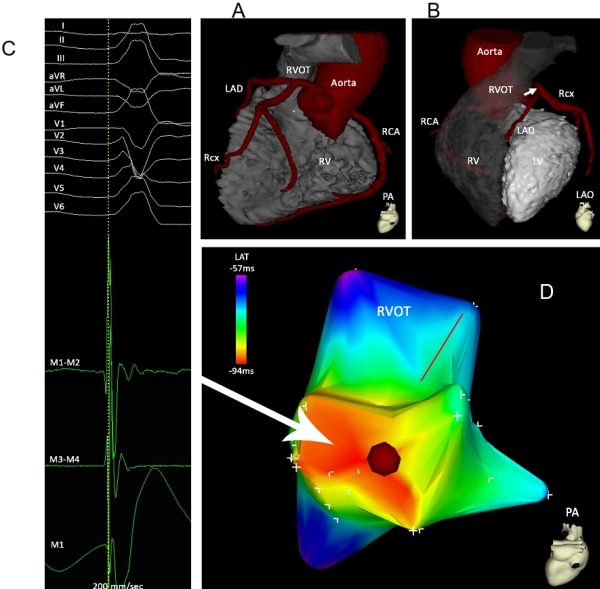
Posterior (Panel A) and left anterior oblique (Panel B) views of the CT scan images of aorta and coronary arteries, RVOT, left and right ventricle to illustrate the spatial relationship between the RVOT, LVOT and the coronary arteries. Please note the close vicinity between the posterior RVOT and the left main (arrow). Panel C: Local bipolar electrograms recorded from the distal (M1-M2), and proximal (M3-M4) electrode pairs and unipolar signal (M1) of the ablation catheter and 12-lead surface ECG of right ventricular outflow tract arrhythmia (sweep speed 200 mm/s). A single radiofrequency application at the recording site abolished the ventricular arrhythmias. Panel D: Posterior view of electroanatomic activation map of the RVOT. Activation time is color coded according to the bar. The red dot indicates the successful ablation site.

Ablation based on activation mapping and/or pace-mapping is considered the favored technique for eliminating idiopathic VT/PVC arising from the RVOT. Systematic point-by-point activation mapping is the initial preferred technique in the presence of sustained tachycardia or frequent PVCs [31,55-57]. The use of a three-dimensional electroanatomical (EAM) systems can assist in relating the anatomy to the mapping data [49,55,57-63] and may facilitate mapping and ablation (Figure 6D). New imaging tools to guide mapping, such as rotational angiography have been recently described [64]. In a small study, performed in 8 patients, the anatomical details provided by three-dimensional rotational angiography were found to be qualitatively superior to the 3D electroanatomical reconstruction using the CARTO system (Biosense Webster, DiamondBar, CA, USA) with comparable procedural characteristics including fluoroscopic exposure [64].

If spontaneous PVCs or VT are absent, programmed stimulation and burst pacing should be performed, and often, catecholamine infusion is required to facilitate induction. Occasionally, epinephrine or phenylephrine may be more effective than isoproterenol. In some patients arrhythmia may manifest after isopreterenol infusion is discontinued [65].

All antiarrhythmic medications should be discontinued for at least five half-lives before the procedure. Deep sedation may also result in non-induciblity of RVOT tachycardia and should be avoided.

## Activation mapping

**Figure 7:**
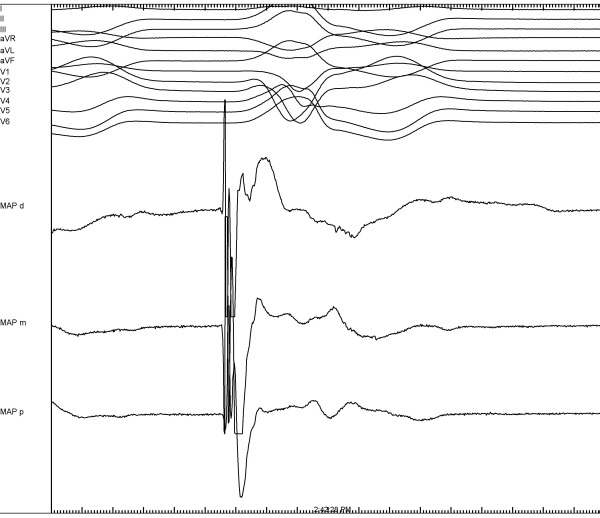
Local bipolar electrograms recorded from the distal (MAPd), mid (MAPm), and proximal (MAPp) electrode pairs of the ablation catheter and 12-lead surface ECG of idiopathic right ventricular outflow tract arrhythmias (sweep speed 200 mm/s). The distal and mid electrode pairs are simultaneously activated, showing a rapid deflection in opposite directions of the initial part of the bipolar electrograms defined as reversed polarity.

During activation mapping the earliest bipolar activity and recording of a local unipolar QS pattern are used to identify the site of origin. Earliest local ventricular activation recorded from the mapping catheter should precede the onset of the surface QRS complex by 10 to 60 ms [56,65]. An unipolar QS pattern with rapid intrinsic deflection demonstrates a high sensitivity for successful ablation sites, but may also be recorded at unsuccessful sites up to 11 mm from the site of origin [66-68]. A newly proposed approach from our centre combines local bipolar activation time with the recording of reversed polarity. The presence of reversed polarity was evaluated from the bipolar electrograms recorded from the distal (M1-M2) and mid (M2-M3) electrode pairs of the mapping catheter and was defined as a rapid simultaneous deflection in opposite direction of the initial part of the bipolar electrograms occurring before the onset of the VA QRS (Figure 7) [66]. This novel approach resulted in a high negative predictive value as well as in a high positive predictive value for identifying a successful ablation site. Conventional mapping based on bipolar activity and an unipolar QS configuration had a comparable negative predictive value but a poor positive predictive value.

If mapping in the RVOT is not successful, the pulmonary artery should be explored. The local bipolar recording at the successful ablation site may demonstrate high frequency, low-voltage signals preceding the main local ventricular activation. Discrete potentials can be recorded from the pulmonary artery preceding the QRS complex during ventricular tachycardia and following the QRS complex in sinus rhythm [45].

## Pace mapping

**Figure 8:**
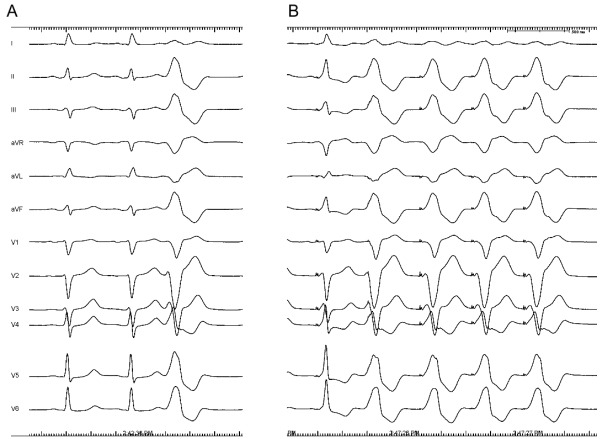
12 lead ECG of the spontaneous PVCs (panel A) and the 12 lead ECG of the paced QRS (3-5 beats, panel B) are shown. Please, note the 12/12 match between the clinical arrhythmia and the paced morphology.

Pace mapping is useful in the absence of frequent PVCs or if tachycardia is not reproducible inducible. It should be performed at the VT cycle length or at a cycle length similar to the coupling interval of ventricular ectopy. Pacing at faster cycle lengths or shorter coupling intervals may lead to rate-dependent changes in QRS morphology [69,70]. The ideal pace-map is an identical QRS pattern in all 12 surface ECG leads (12/12 match) between the clinical arrhythmia and the paced QRS morphology (Figure 8) [57,71]. Pacing should be performed at stimulus strengths only slightly higher than the diastolic threshold to avoid capture over a large area which is likely to reduce accuracy. In the absence of a good pace map, the catheter should be carefully repositioned within the area of interest.

However, it is important to consider that the spatial resolution of pace-mapping was found to be inferior to that of activation mapping [56]. A good pace-map could be obtained at sites with a mean distance of 7.3±5mm from the effective ablation site. In addition, in 18% of the patients the pace-map at the successful ablation site was poor. The latter was explained by a site of origin located deep to the endocardium with conduction through preferential fibers connecting the site of origin to the endocardium.

## Indications and contraindications for an ablative approach

According to the current guidelines [1], catheter ablation is recommended in patients with severely symptomatic ventricular ectopy/tachycardia or if antiarrhythmic drug therapy remains ineffective, is poorly tolerated or not desired. In addition, catheter ablation is indicated for recurrent sustained polymorphic VT and VF that is refractory to antiarrhythmic therapy when there is a suspected trigger that can be targeted for ablation. Finally, catheter ablation is recommended for patients with frequent PVCs, nonsustained VTs, or VT that is presumed to cause ventricular dysfunction. Contraindications to catheter ablation are asymptomatic ventricular ectopy or infrequent nonsustained tachycardia or if OT tachycardia is due to reversible causes [1].

## Catheter choice and energy settings

Ablation within the RVOT may be performed using a conventional solid tip or an irrigated tip catheter. If an irrigated tip catheter is used, power should be carefully titrated (up to 35W) with a maximum temperature of 45ºC. Termination is expected to occur within 10 seconds of radiofrequency current energy delivery; otherwise mapping should be continued. Acceleration of the tachycardia during ablation, followed by gradual slowing or abrupt termination may be observed [31]. Similarly, application of radiofrequency (RF) current during sinus rhythm at sites of the origin of the VA may also lead to induction of repetitive responses or VA with similar morphology as seen during spontaneous RVOT VA. The changing patterns of repetitive ventricular response (slowing and/or disappearance) have been shown to be consistent with successful RF ablation [72].

Recently, the efficacy, safety, and feasibility of new techniques for RVOT VA ablation, such as remote-controlled magnetic navigation system, have been reported. This technique may have advantages with respect to reduction in the operator's and patient's radiation exposure and a lower incidence of catheter-induced PVCs [73].

## Complications

Complications are rare and they are usually related to damage of structures in close anatomical proximity to the site of ablation. The distance from the leftward posterior aspect of the RVOT to the left main coronary artery is 4.1±1.9 mm (Figure 6A,B) [74]. Therefore, ablation along the posterior RVOT may result in damage to the left main coronary artery. Other risks include cardiac perforation resulting in tamponade, complications related to vascular access or damage to the His bundle with consecutive heart block.

## Procedural outcome following catheter ablation of OT tachycardias

The acute success rate for ablation of RVOT tachycardia ranges from 75 to 100% with a low recurrence rate of 5% (Table 2). Wen et al. [80] described a poor pace-map score of <10/12, reliance on pace-map only in patients with no sufficient ventricular tachycardia for identification of ablation sites, and a later local activation time at ablation site as predictors of recurrence. Flemming et al.[82] found that the best discriminator of outcome was the QRS duration in lead V2 with a likelihood of successful ablation with an endocardial approach in the right ventricular outflow tract of 95% when VT/bigeminy QRS duration was ≥ 160 ms compared with only 54% if the QRS duration in V2 was <160 ms in duration. However, in a previous study [79], an unsuccessful outcome was associated with a delta wave-like onset of the QRS complex during ventricular tachycardia, suggesting that a slow onset of the QRS complex may indicate an intramural or epicardial origin of the ventricular tachycardia. Other factors predictive of unsuccessful ablation were more than one induced VT morphology and a match between the clinical VT and pace-map in less than 11 of 12 leads.

## Conclusions

Idiopathic VA occurs in individuals with no structural disease and predominantly originate from the RVOT. These arrhythmias have a focal origin with triggered activity due to delayed after-depolarizations as the main underlying mechanism. The typical QRS morphology during idiopathic focal RVOT VT shows LBBB configuration with an inferior (right or left) axis.

Precise localization for ablation is ideally guided by activation mapping, although pace mapping, or a combination of both methods may be applied in case of infrequent arrhythmia. RF catheter ablation of RVOT-VT is a safe treatment option with an acute success > 90% and a low risk of complications, and could be considered first-line therapy in selected patients.

## Figures and Tables

**Table 1:**
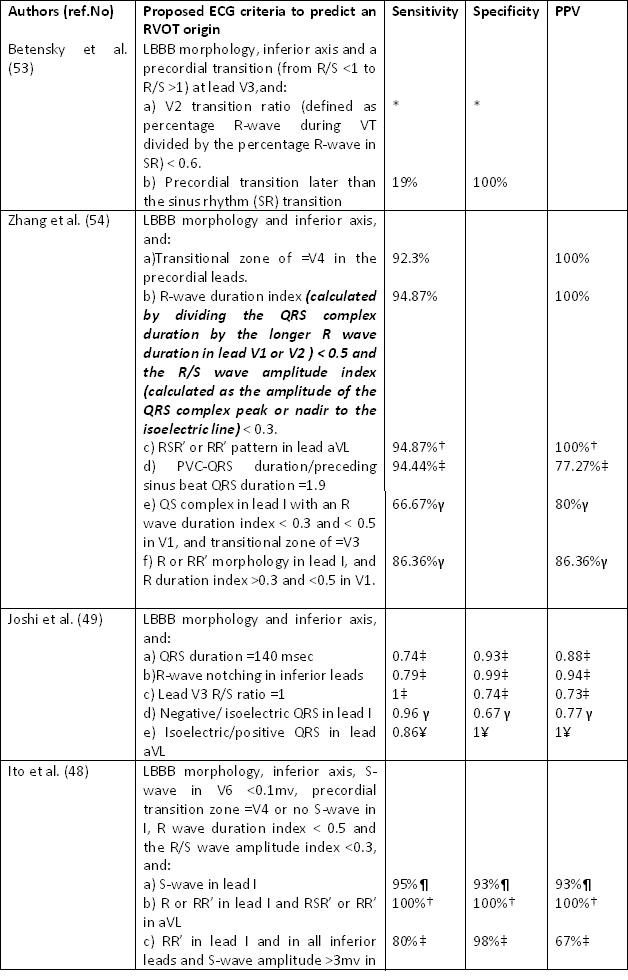
Proposed Electrocardiographic criteria to predict the origin of outflow tract ventricular tachycardia.

LBBB: Left bundle branch block; PPV: positive predictive value * V2 transition ratio ≥ 0.6 predicts an LVOT origin with a sensitivity of 95% and specificity of 100%. †  Values for VT/PVCs originated from the His bundle region ‡ Values for VT/PVCs originated from the free wall γ Values VT/PVCs from anterior site Â Values VT/PVCs from caudal site (>2 cm from Pulmonary vein) ¶ Values VT/PVCs from septum of the RVOT

**Table 2:**
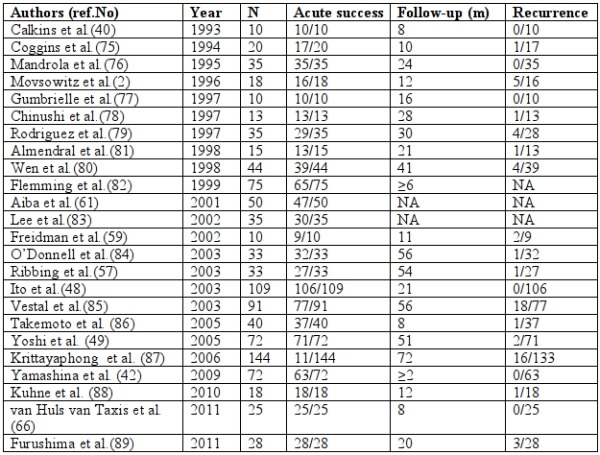
Outcome of Radiofrequency Catheter Ablation in Patients with Idiopathic Right Ventricular Outflow Tract Tachycardia.
